# NAMPT/SIRT2-mediated inhibition of the p53-p21 signaling pathway is indispensable for maintenance and hematopoietic differentiation of human iPS cells

**DOI:** 10.1186/s13287-021-02144-9

**Published:** 2021-02-05

**Authors:** Yun Xu, Masoud Nasri, Benjamin Dannenmann, Perihan Mir, Azadeh Zahabi, Karl Welte, Tatsuya Morishima, Julia Skokowa

**Affiliations:** 1 Department of Hematology, Oncology, Clinical Immunology and Rheumatology, University Hospital Tübingen, Tübingen, Germany; 2 University Children’s Hospital Tübingen, Tübingen, Germany; 3 present address: International Research Center for Medical Sciences, Kumamoto University, Kumamoto, Japan

**Keywords:** iPSC maintenance, Hematopoietic differentiation of iPSCs, NAMPT/SIRT2 pathway, p53 deacetylation, p21 activation

## Abstract

**Background:**

Nicotinamide phosphoribosyltransferase (NAMPT) regulates cellular functions through the protein deacetylation activity of nicotinamide adenine dinucleotide (NAD^+^)-dependent sirtuins (SIRTs). SIRTs regulate functions of histones and none-histone proteins. The role of NAMPT/SIRT pathway in the regulation of maintenance and differentiation of human-induced pluripotent stem (iPS) cells is not fully elucidated.

**Methods:**

We evaluated the effects of specific inhibitors of NAMPT or SIRT2 on the pluripotency, proliferation, survival, and hematopoietic differentiation of human iPS cells. We also studied the molecular mechanism downstream of NAMPT/SIRTs in iPS cells.

**Results:**

We demonstrated that NAMPT is indispensable for the maintenance, survival, and hematopoietic differentiation of iPS cells. We found that inhibition of NAMPT or SIRT2 in iPS cells induces p53 protein by promoting its lysine acetylation. This leads to activation of the p53 target, p21, with subsequent cell cycle arrest and induction of apoptosis in iPS cells. NAMPT and SIRT2 inhibition also affect hematopoietic differentiation of iPS cells in an embryoid body (EB)-based cell culture system.

**Conclusions:**

Our data demonstrate the essential role of the NAMPT/SIRT2/p53/p21 signaling axis in the maintenance and hematopoietic differentiation of iPS cells.

**Supplementary Information:**

The online version contains supplementary material available at 10.1186/s13287-021-02144-9.

## Highlights

- NAMPT regulates proliferation and survival of iPS cells via SIRT2
- SIRT2 deacetylates p53 leading to inhibition of p21 in iPS cells
- NAMPT and SIRT2 are important for the EB-based hematopoietic differentiation of iPS cells

## Introduction

Understanding the mechanisms underlying the maintenance and hematopoietic development of induced pluripotent stem (iPS) cells is essential for the establishment of efficient protocols for iPS cell generation, ex vivo blood cell formation, and the identification of new treatment options for benign and oncogenic hematological disorders.

Although protocols for iPS cell generation are well established, there is still room for improvement in terms of the efficient generation of high-quality iPS cells. Understanding the mechanisms that maintain pluripotency and proliferation of iPS cells will be helpful in screening for highly pluripotent, high-quality iPS cells as well as improving protocols for the large-scale generation and maintenance of high-quality iPS cells.

Establishment of protocols for in vitro hematopoietic differentiation of embryonic stem (ES) cells and iPS cells has enabled the identification of a plethora of extrinsic and intrinsic factors essential for the regulation of blood cell differentiation at different developmental stages, starting from very early stages of mesodermal specification and the generation of early hemogenic progenitors [1, 2]. Diverse, crucial hematopoietic transcription factors are deregulated in human bone marrow failure syndromes and leukemia [3–5]. Regulation of these key factors can take place at the transcriptional or translational level, but there is also growing evidence for post-translational modifications, such as phosphorylation or de-/acetylation, in the regulation of protein functions [6, 7]. For instance, protein deacetylation triggered by NAMPT (nicotinamide phosphoribosyltransferase) and downstream NAD^+^-dependent sirtuins (SIRT) is important for myeloid differentiation and leukemogenic transformation of hematopoietic cells through regulation of the CCAAT/enhancer-binding proteins C/EBPα and C/EBPß, the serine/threonine kinase AKT, the tumor-suppressor p53, and the forkhead box transcription factor FOXO3 [8–12]. In these studies, SIRT1 and SIRT2, members of the SIRT family of NAD^+^-dependent class III histone deacetylases [13], were found to activate target proteins in hematopoietic cells upon NAMPT activation. It has been demonstrated that a SIRT1 deficiency compromises hematopoietic differentiation of mouse ES cells and embryonic and adult hematopoiesis in the mouse [14]. However, the role of NAMPT and SIRT2 during maintenance and myeloid differentiation of iPS cells is largely unknown. Identification of specific selective inhibitors of NAMPT and SIRT2 [15, 16] has made it possible to evaluate the specific roles of each of these factors in different physiological and pathological processes. We recently identified important roles of NAMPT and SIRT2 during early stages of hematopoietic differentiation of iPS cells using a feeder-free, serum-free monolayer-based differentiation protocol [17]. However, we did not study the granulocytic differentiation of iPS cells using this method.

In the current study, we evaluated the roles of NAMPT and SIRT2 in the maintenance and granulocytic differentiation of iPS cells. We found that NAMPT/SIRT2-mediated deacetylation of p53 is important for iPS cell maintenance through deactivation of p53 and subsequent suppression of p21.

## Material and methods

### Cell culture

Healthy donor-derived human iPS cells (hiPSCs, hCD34-iPSC16) [18] were provided by Dr. Nico Lachmann and Dr. Thomas Moritz (Hannover Medical School, Hannover, Germany). This hiPSC line was maintained on Geltrex LDEV-Free Reduced Growth Factor Basement Membrane Matrix (Cat Nr. A1413302, Thermo Fisher Scientific)-coated cell culture plates in Stemflex medium with 10% Stemflex Supplement (Cat Nr. A3349401, Thermo Fisher Scientific) and 1% penicillin/streptomycin. The medium was changed every day or every second day. Cells were passaged every 5 or 6 days in 1:10 or 1:15 ratios depending on their density.

### Treatment of iPS cells with FK866 or AC93253

5 × 10^4^ hiPSCs/well were seeded into one well of a 6-well plate, were kept in maintenance for 48 h, and were then treated with different doses of FK866 (Cat Nr. F8557-25MG, Sigma-Aldrich) or AC93253 (Cat Nr. A9605-10MG, Sigma-Aldrich). The corresponding concentration of dimethylsulfoxide (DMSO; Sigma-Aldrich) was used as vehicle control. After 48 h cells were collected for further analysis.

### Flow cytometry analysis

To assess the pluripotency of iPS cells, the antibodies TRA1-60-PE (Cat Nr. MA1-023-PE, eBioscience) and SSEA4-FITC (Cat Nr. 560126, BD biosciences, BD) were used. Dead cells were excluded from the analysis by 4′,6-diamidino-2-phenylindole (DAPI; 1μg/ml) (Cat Nr. D3571, Thermo Fisher Scientific) staining. For detection of hematopoietic progenitor cells, the antibodies CD33-BV421 (Cat Nr. 366622, BioLegend, BL), CD34-PE-Cy7 (Cat Nr. 343615, BL), KDR-AF647 (Cat Nr. 359909, BL), CD43-PE (Cat Nr. 343204, BL), CD41a-FITC (Cat Nr. 303703, BL), CD235a-FITC (Cat Nr. 349103, BL), CD45-BV510 (Cat Nr. 103138, BL) and 7-AAD (Cat Nr. 420404, BL) were used as an “early-stage” multicolor hematopoietic cell panel. For the detection of mature myeloid cells, the antibodies CD15-PE (Cat Nr. 301905, BL), CD16-FITC (Cat Nr. 302005, BL), CD14-APC-H7 (Cat Nr. 367117, BL), CD45-BV510 (Cat Nr. 103138, BL), CD33 BV-421 (Cat Nr. 366622, BL) and 7-AAD (Cat Nr. 420404, BL) were used as a “late-stage” multicolor myeloid differentiation panel. Anti-mouse IgGk beads were used for compensation. Antibodies and beads for flow cytometry were purchased from BD Biosciences unless otherwise indicated. Samples were analyzed using a FACS Canto II flow cytometer (Becton-Dickinson) and FlowJo software (FLOWJO, LLC, Ashland, OR).

### RNA isolation and qRT-PCR

RNA was isolated using the RNeasy mini kit (Qiagen), and cDNA was prepared from 500 ng RNA by oligo primer using the Omniscript-RT kit (Qiagen). All procedures were performed following the manufacturers’ instructions. Quantitative polymerase chain reaction (qPCR) was performed using LightCycler® 480 SYBR Green I Master (Roche Applied Science). Real-time PCR detection was performed using a LightCycler 480 Real-Time PCR System (Roche Applied Science). Quantification of target gene expression was conducted in comparison to the reference GAPDH gene expression and depicted as ∆∆Ct relative to GAPDH. Primer sequences are shown in Table S1.

### Lentivirus-mediated gene knockdown in hiPSCs

HEK293T cells were used for lentivirus production. On the day before transfection, 8× 10^6^ HEK293T cells were plated in each T75 flask. Cells were co-transfected with target shRNA expression vector (NAMPT shRNA in pRRL.PPT.SF.i2RFP, SIRT2 shRNA in pRRL.PPT.SF.i2GFP, p53 shRNA in pRRL.PPT.SF.i2YFP) or control vector (pRRL.PPT.SF.i2GFP, pRRL.PPT.SF.i2RFP, or pRRL.PPT.SF.i2YFP), psPAX2 packaging vector (#12260, Addgene), and pMD2.G envelope vector (#12259, Addgene) using TransIT®-LT1 Transfection Reagent (#MIR2305, Mirus Bio LLC). Oligonucleotide sequences for shRNA are available upon request. Lentivirus-containing supernatants were harvested at 48 h after transfection, passed through a 0.22-μm filter, and incubated with Lenti-X™ Concentrator (#631232, Takara Clonetech) overnight at 4 °C. After centrifugation, the virus pellet was resuspended with complete DMEM medium and titrated by FACS.

For knockdown of NAMPT, SIRT2, or p53, hiPS cells were seeded in a 6-well plate (1 × 10^5^ cells/well) 24 h before transduction. Cells were transduced by incubation and centrifugation with lentiviral supernatant at a multiplicity of infection (MOI) 40. After 72 h incubation at 37 °C, transduction efficiency was quantified by qPCR and western blot.

### Western blot analysis

Whole-cell lysates were obtained by lysing equal numbers of cells with 3× laemmli buffer (30% glycerol, 6% SDS, 7.5% β-Mercaptoethanol, 0.75% Bromphenol blue in 200 nM Tris-HCL [pH 6.8]), which were subsequently heated at 95 °C for 5 min and spun down. Proteins were separated by SDS-PAGE and transferred to nitrocellulose membranes (GE healthcare life sciences). The WB membranes were blocked with 5% non-fat dry milk-TBST (10 mM Tris-HCL [pH 8.0], 150 mM NaCl, 0.1% Tween 20) for 1 h at room temperature. Primary antibodies were incubated overnight at 4 °C. After washing 4 times for 5 min with TBST, membranes were incubated with secondary antibodies for 1 h at room temperature. The protein bands were detected using Pierce ECL Western Blotting substrate (Thermo Fisher Scientific) or Luminata Forte Western HRP substrate (Millipore, Billerica, MA) and visualized by exposure to X-ray film (GE healthcare life sciences). The following antibodies were used: rabbit monoclonal antibody to GAPDH (Cat Nr. 2118, Cell Signaling Technology), rabbit monoclonal antibody to p21 (Cat Nr. 2947s, Cell Signaling Technology), mouse monoclonal antibody to p53 (Cat Nr. sc-126, Santa Cruz Biotechnology), rabbit monoclonal antibody to acetyl-p53 (Lys382) (Cat Nr. 2525, Cell Signaling Technology), rabbit polyclonal antibody to NAMPT (Cat Nr. PAB17046, Abnova).

### Assessment of cell proliferation with Incucyte® S3 live-cell analysis system

2 **×** 10^4^ hiPSCs were seeded in Geltrex-coated cell culture plates in Stemflex medium with 10% Stemflex Supplement (Cat Nr. A3349401, Thermo Fisher Scientific). The cells were incubated in the medium supplemented with FK866 1 nM/2 nM, AC93253 50 nM/100 nM or DMSO at the corresponding concentrations in IncuCyte Live Cell Analysis System (Essen Bio) at 37 °C, 5% CO_2_. The cell growth images were recorded every 6 h and analyzed by IncuCyte S3 Software (Essen Bio).

### Cell cycle analysis

For cell cycle analysis, iPS cells were incubated with 1 mM of BrdU for 30 min and BrdU uptake was quantified using APC BrdU Flow Kit (Cat Nr. 557892, Becton-Dickinson, Franklin Lakes, NJ, USA). Samples were analyzed using a FACS Canto II flow cytometer (Becton-Dickinson) and FlowJo software (FLOWJO, LLC, USA).

### Assessment of apoptosis

Apoptosis was analyzed using FITC Annexin V Apoptosis Detection Kit (Cat Nr. 556547, Becton-Dickinson) following the manufacturer’s instructions. Samples were analyzed using a FACS Canto II flow cytometer (Becton-Dickinson) and FlowJo software (FLOWJO, LLC).

### Embryoid body (EB)-based hematopoietic differentiation of hiPSCs

hiPS cells were kept in maintenance on Geltrex-coated plates for 5 days until confluency. iPS cells were dissociated by PBS/EDTA (0.02%) for 5–7 min. EB induction was achieved via Spin EBs (20.000 cells/EB) in 96-well plates using APEL serum-free differentiation medium (Stemcell Technologies) supplemented with bFGF (20 ng/μl) and ROCK Inhibitor (R&D). After 24 h, BMP4 (40 ng/μl) was added to the culture to induce mesodermal differentiation. After 2 days, EBs were plated on Geltrex-coated 6-well plates (10 EBs/well) in hematopoietic stem cell differentiation medium (APEL medium supplemented with 40 ng/μl VEGF, 50 ng/μlSCF, and 50 ng/μl IL-3). After 3 days, medium was changed to the neutrophil differentiation medium (APEL medium supplemented with 50 ng/μl IL3 and 50 ng/μl G-CSF). DMSO, FK866 (1 nM and 2 nM), or AC93253 (50 nM and 100 nM) were added to the culture medium starting at day 3 of culture. Medium with DMSO, FK866, or AC93253 was exchanged every 3 days. Hematopoietic cells appeared on days 12–14 of culture. They were harvested for various analyses on days 18 and 25. All cytokines were purchased from R&D System unless otherwise indicated. Cell morphology was evaluated on cytospin preparations of suspension hematopoietic cells generated on day 25 of culture. For this, 2 × 10^4^ cells were centrifuged on the cytospin centrifuge at 400 rpm for 4 min. Cytospin slides were stained with Wright-Giemsa stain using the Hema-Tek slide stainer (Ames).

### Three-germ-layer differentiation assay

The three-germ-layer differentiation of hiPS cells was performed using STEMdiff^TM^ Trilineage Differentiation Kit following the manufacturer’s instructions (Cat Nr. 05230, Stemcell Technologies). In some experiments, cell culture medium was supplemented with FK866 1 nM, AC93253 50 nM, or DMSO. For ectoderm lineage, 4 × 10^5^ hiPS cells per well were plated in 24-well plate on day 0. After 24 h, the culture medium was changed from Stemflex medium to STEMdiff^TM^ Trilineage Ectoderm Medium (Cat Nr. 05231, Stemcell Technologies). The medium was changed daily until day 7. 1 × 10^5^ hiPS cells per well for mesoderm lineage and 4 × 10^5^ hiPS cells per well for endoderm lineage were plated in 24-well plates on day 2. The cells were supplemented daily with STEMdiff™ Trilineage Mesoderm Medium (Cat Nr. 05232, Stemcell Technologies) and STEMdiff™ Trilineage Endoderm Medium (Cat Nr. 05233, Stemcell Technologies) with inhibitors or DMSO starting from day 3 till day 7.

Cell differentiation was analyzed on day 7 using the Human Three Germ Layer 3-color Immunocytochemistry Kit according to the manufacturer’s instructions (Cat Nr. SC022, R&D Systems). Cells were fixed in PBS containing 4% paraformaldehyde (Cat Nr. 158127, Sigma-Aldrich) and blocked with PBS containing 10% normal donkey serum (Cat Nr. D9663, Sigma-Aldrich), 0.3% Triton™ X-100 (Cat Nr. 93443, Sigma-Aldrich), and 1% BSA (Cat Nr. A2058, Sigma-Aldrich). After that, cells were incubated with conjugated antibodies corresponding to the cell lineage of interest: Otx-2 for ectoderm, Brachyury for mesoderm, and GATA-4 for endoderm. After 3 h incubation at room temperature in the dark, cells were washed and kept with PBS containing 1% BSA and DAPI (1:1000 dilution). Images were taken on ZEISS Apotome microscope.

### Statistical analyses

Statistical analyses were conducted using Student’s *t* test or Boost Ratio [19] Statistical significance was taken to be *p* < 0.05.

## Results

### Inhibition of NAMPT or SIRT2 suppresses growth and induces cell cycle arrest and apoptosis in human iPS cells

**Fig. 1:**
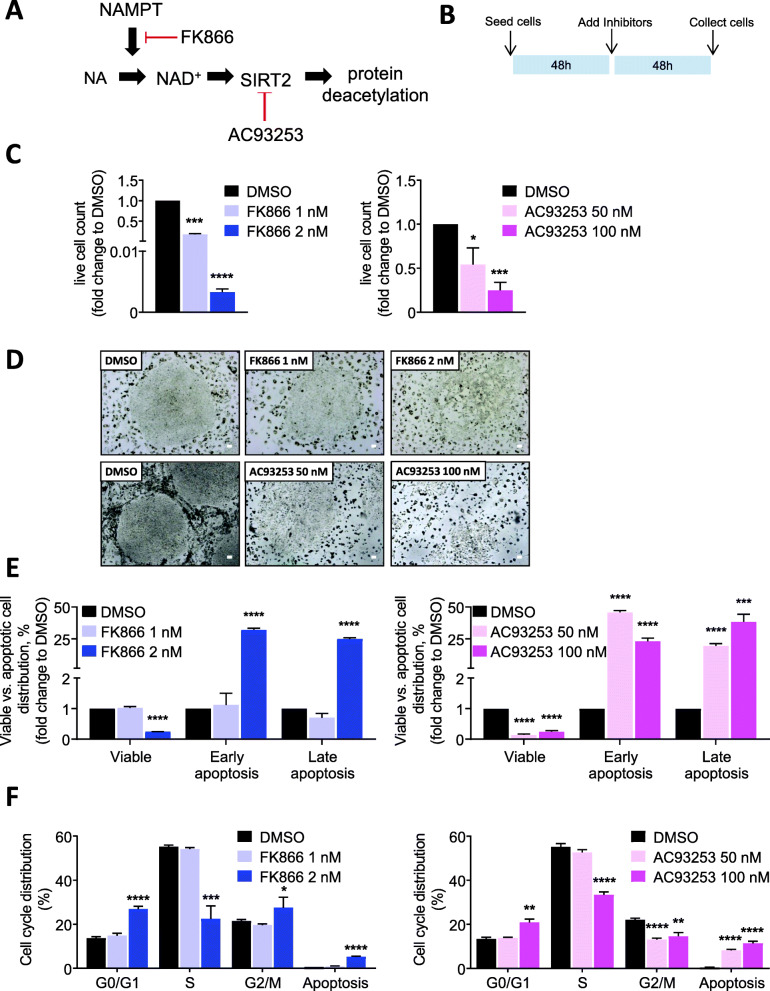
Inhibition of NAMPT or SIRT2 suppresses the proliferation of human iPS cells by enhanced apoptosis and cell cycle arrest. **a** Schematic of the NAMPT-NAD^+^-SIRT2 pathway. NAMPT is the rate-limiting enzyme that converts nicotinamide (NA) into NAD^+^ that subsequently activates the NAD^+^ dependent protein deacetylase, SIRT2. Specific small molecule inhibitors for NAMPT (FK866) and SIRT2 (AC93253) are depicted in red. **b** 5 × 10^4^ human iPS cells were seeded on a Geltrex-coated 6-well plates, as described in MM. After 48 h of culture, different doses of FK866 or AC93253 were added to the culture medium. DMSO was used as vehicle control. Cell numbers were counted after 48 h of culture. **c** Numbers of viable iPS cells treated with FK866 (left) or AC93253 (right) were quantified using trypan blue dead cell exclusion. DMSO was used as vehicle control. Fold change differences of live cells relative to DMSO-treated cells are shown. Data represent means ± SD from two independent experiments, each in triplicates (*p < 0.05, ***p < 0.001, ****p < 0.0001). **d** Representative images of human iPS cells treated with DMSO, FK866, or AC93253 for 48 h. Scale bars: 50 μm. **e** Analysis of apoptosis of FK866 (left) or AC93253 (right)-treated human iPS cells using Annexin V assay. DMSO was used as vehicle control. Diagrams show the fold change differences in the percentage of each cell fraction (early apoptosis, late apoptosis, viable cells) relative to DMSO-treated cells. Data represent means ± SD from two independent experiments, each in triplicates (***p < 0.001, ****p < 0.0001, compared to DMSO-treated cells). **f** Analysis of cell cycle of FK866 (left) or AC93253 (right)-treated human iPS cells using BrdU assay. DMSO was used as vehicle control. Data represent means ± SD from two independent experiments, each in triplicates, (*p < 0.05, **p < 0.01, ***p < 0.001, ****p < 0.0001)

We tested the in vitro effect of FK866 (a specific inhibitor of NAMPT) and AC93253 (a highly selective SIRT2 inhibitor) on the growth of human iPS cells (Fig. 1a, b). We found that treatment with FK866 or AC93253 caused a concentration-dependent decrease in the absolute number of iPS cells (Fig. 1c). These results are in line with reduced NAD^+^ levels in FK866-treated cells (Fig. S1A). Morphologically, iPS cells treated with FK866 or AC93253 failed to form compact colonies, compared with control cells treated with DMSO (Fig. 1d). To explore the mechanism underlying the defect in iPS cell proliferation, we measured apoptosis and assessed cell cycle distribution using Annexin V and BrdU assays after treating cells with different concentrations of FK866 (1 and 2 nM), AC93253 (50 and 100 nM), or DMSO (vehicle control). We found that, compared with control cells, treatment with FK866 (2 nM) or AC93253 (50 and 100 nM) induced early and late apoptosis in iPS cells (Fig. 1e, Fig. S2A). Additionally, inhibition of NAMPT or SIRT2 caused cell cycle arrest in G0/G1 phase in iPS cells (Fig. 1f, Fig. S2B).

**Fig. 2:**
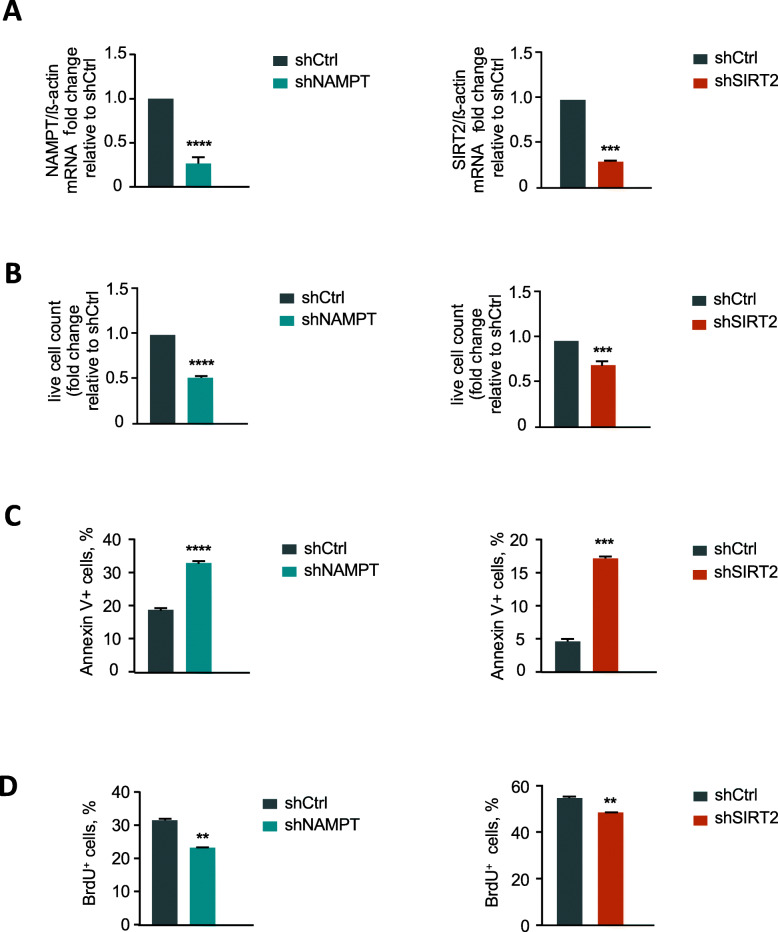
Effects of NAMPT or SIRT2 knockdown on the proliferation and survival of human iPS cells. **a** mRNA expressions of NAMPT (left) and SIRT2 (right) in iPS cells transduced with shNAMPT (left) or shSIRT2 (right) LV were evaluated. iPS cells transduced with shCtrl LV was used as control. Fold changes relative to control are shown (***p < 0.001, ****p < 0.0001). **b** Cell growth of iPS cells transduced with shNAMPT (left) or shSIRT2 (right) lentivirus (LV) were quantified using trypan blue dead cell exclusion. iPS cells transduced with shCtrl LV was used as control. Fold changes relative to control are shown (***p < 0.001, ****p < 0.0001). **c** Apoptosis was analyzed in shNAMPT (left) or shSIRT2 (right) LV-transduced iPS cells using Annexin V assay. iPS cells transduced with shCtrl LV were used as control. Diagrams show the percentage of Annexin V-positive apoptotic cells (**p < 0.01, ***p < 0.001, ****p < 0.0001). **d** Cell cycle was analyzed in shNAMPT (left) or shSIRT2 (right) LV-transduced iPS cells using BrdU assay. iPS cells transduced with shCtrl LV was used as control. Diagrams show the percentage of cells in S phase (**p < 0.01)

Similar results were observed by inhibiting NAMPT or SIRT2 by means of lentivirus-based transduction of iPS cells with shRNAs specifically targeting human NAMPT or SIRT2, as compared to control shRNA-transduced samples (Fig. 2a–d, Fig. S5A).

### Inhibition of NAMPT or SIRT2 affects the pluripotency of human iPS cells

**Fig. 3:**
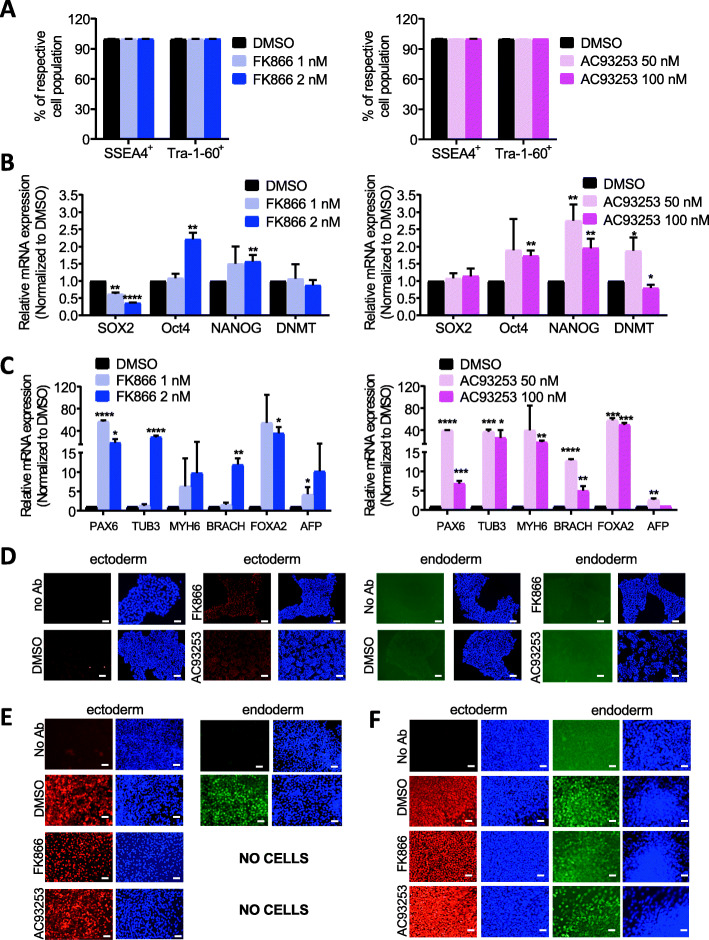
Effect of NAMPT or SIRT2 inhibition on the iPS cell pluripotency. **a** Analysis of the expression of the pluripotent stem cell surface markers, SSEA4 and Tral-1-60, on FK866 (left) or AC93253 (right)-treated human iPS cells using FACS. DMSO was used as vehicle control. Data represent means ± SD from two independent experiments, each in triplicates. **b** mRNA expression of pluripotency genes in human iPS cells treated with FK866, AC93253, or DMSO for 48 h. Fold changes of pluripotency markers relative to DMSO-treated cells are shown. Data represent means ± SD from two independent experiments, each in triplicates (*p < 0.05, **p < 0.01, ****p < 0.0001). **c** mRNA expression of genes specific for the three germ layers in human iPS cells treated with FK866, AC93253, or DMSO for 48 h. Fold changes of markers of differentiation relative to DMSO-treated cells are shown. Data represent means ± SD from two independent experiments, each in triplicates (*p < 0.05, **p < 0.01, ****p < 0.0001). **d** iPS cells were treated with DMSO, 1 nM of FK866, or 50 nM of AC93253 for 48 h with subsequent ICH analysis of the ectodermal and endodermal differentiation, as described in “Material and methods”. Representative images are depicted, no Ab: control staining w/o antibody. **e** Ectodermal and endodermal differentiation of iPS cells in the presence of DMSO, 1 nM FK866, or 50 nM AC93253 was evaluated, as described in “Material and methods”. Representative images are depicted, no Ab: control staining w/o antibody. **f** iPS cells were treated with DMSO, 1 nM FK866, or 50 nM AC93253 for 48 h, after that ectodermal and endodermal differentiation was performed, as described in “Material and methods”, without addition of inhibitors. Representative images are depicted, no Ab: control staining w/o antibody

We further evaluated the effects of NAMPT or SIRT2 inhibition on the pluripotency of human iPS cells. We found that neither SSEA4 and Tra-1-60 protein expression nor alkaline phosphatase staining were not affected by treatment of iPS cells with FK866 or AC93253 compared with DMSO-treated control group (Fig. 3a, Figs. S3A-B, S4A). At the same time, SOX2 mRNA expression in iPS cells was inhibited by FK866 treatment compared with DMSO controls, whereas Oct4 and NANOG mRNA levels were increased by FK866 or AC93253 treatment (Fig. 3b). An analysis of mRNA expression of genes specific for each of three germ layers, ectoderm (Pax6, TUB3), mesoderm (MYH6, BRACH), and endoderm (FOXA2, AFP), revealed that TUB3, BRACH, and AFP were upregulated by treatment with FK866 (2 nM), whereas incubation with AC93253 induced Pax6, BRACH, and FOXA2 expression compared to the vehicle control (Fig. 3c). At the same time, protein expression of endoderm-, mesoderm-, and ectoderm-specific proteins was not affected (Fig. 3d and data not shown).

Interestingly, differentiation of iPSCs into ectoderm and endoderm in the presence of NAMPT or SIRT2 inhibitors resulted in a strong suppression of the endodermal differentiation, but no effects on the ectodermal differentiation (Fig. 3e and data not shown). At the same time, pre-culture of iPSCs with NAMPT or SIRT2 inhibitors for 48 h and subsequent differentiation into endoderm and ectoderm in the absence of inhibitors revealed no differences between studied groups (Fig. 3f and data not shown).

These results indicate that inhibition of NAMPT or SIRT2 in human iPS cells deregulates controlled expression of pluripotency and germ layer genes affecting endodermal, but not ectodermal differentiation. This process is reversible, since endodermal differentiation was not affected upon removal of the inhibitors.

### Inhibition of NAMPT or SIRT2 attenuates mesodermal and neutrophil differentiation of human iPS cells

**Fig. 4:**
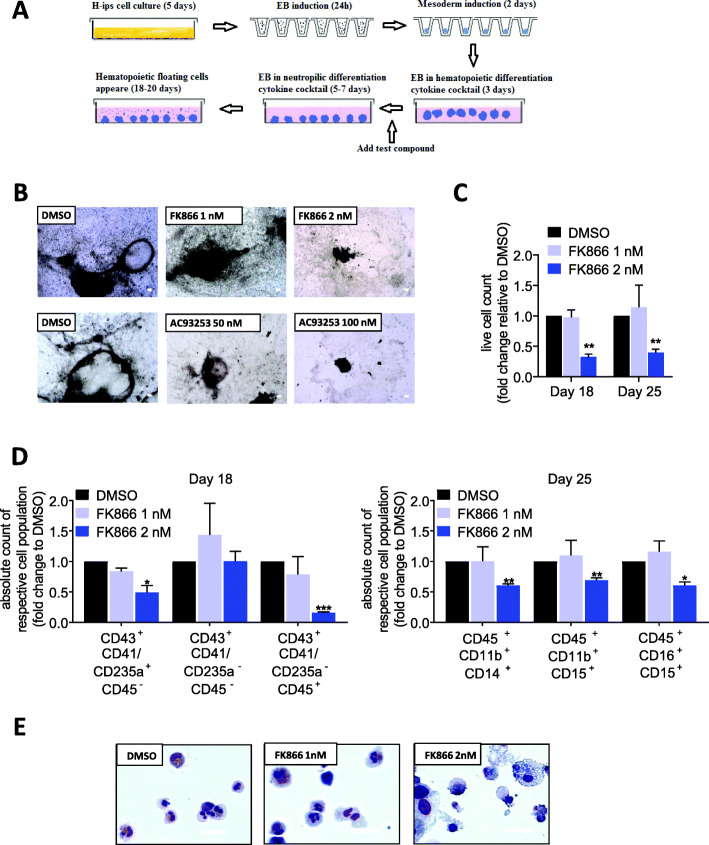
NAMPT or SIRT2 inhibition suppresses hematopoietic differentiation of iPS cells. **a** Work flow of EB-based hematopoietic differentiation of iPS cells in the presence of DMSO, FK866, or AC93253. **b** Representative images of differentiated iPS cells treated with indicated drugs for 25 days. Images were taken using a Nikon Eclipse TS 100 microscope. Scale bars: 50 μm. **c** Number of viable hematopoietic cells generated in the presence of FK866 or DMSO using EB-based hematopoietic differentiation protocol at indicated time points, as assessed using trypan blue dead cell exclusion assay. Fold changes relative to DMSO-treated cells are shown. Data represent means ± SD from two independent experiments, each in duplicates (**p < 0.01). **d** Flow cytometry analysis of suspension cells harvested from EBs culture on day 18 and day 25 of differentiation for iPS cells treated with DMSO or FK866. Fold change difference of the absolute numbers of each cell fraction relative to DMSO are shown. Data represent means ± SD from two independent experiments, each in duplicates (*p < 0.05, **p < 0.01, ***p < 0.001). **e** Representative cytospin images of suspension cells derived from differentiated human iPS cells treated with DMSO or FK866 on day 25 of culture. Scale bars: 400 μm

We further evaluated the effects of NAMPT and SIRT2 inhibitors on the mesodermal and hematopoietic/granulocytic differentiation of iPS cells. We recently described a novel mechanism for the granulocytic differentiation of hematopoietic stem cells by NAMPT-mediated protein deacetylation [8]. We therefore evaluated the role of NAMPT and SIRT2 in the neutrophilic differentiation of iPS cells using an embryoid body (EB)-based culture system (Fig. 4a). We found that cells treated with 2 nM FK866 produced significantly fewer hematopoietic cells compared with control cells. Treatment with AC93253 (50 or 100 nM) completely suppressed hematopoietic differentiation. In contrast, treatment with 1 nM of FK866 had almost no effect on differentiation (Fig. 4b, c). We also analyzed these differentiated cells by flow cytometry and found a decrease in erythro-megakaryocytic progenitors (CD43^+^CD41a^+^CD235a^+^CD45^−^) [20] and myeloid-committed multilineage progenitors (CD43^+^CD41a^−^CD235a^−^CD45^+^) [20] in cells treated with 2 nM FK866 compared with cells treated with DMSO or 1 nM FK866, whereas multilineage progenitors with lymphoid potential (CD43^+^CD41a^−^CD235a^−^CD45^−^) [20] were unchanged. We also observed a decrease in monocytes (CD45^+^CD11b^+^CD14^+^), immature neutrophils (CD45^+^CD11b^+^CD15^+^), and mature neutrophils (CD45^+^CD16^+^CD15^+^) in response to treatment with 2 nM FK866, but not 1 nM FK866 or DMSO (Fig. 4d, Figs. S6A, S7A). In addition, an evaluation of cytospin slides showed an increased number of immature myeloid cells as a consequence of 2 nM FK866 treatment (Fig. 4e). These findings are in line with reduced levels of intracellular NAD^+^ in suspension EB-derived hematopoietic cells and in adherent EB cells assessed on day 29 of culture (Fig. S8A). Collectively, these data suggest an important role for NAMPT, SIRT2, and NAD^+^ in the regulation of mesodermal and granulocytic differentiation of iPS cells.

### Inhibition of NAMPT or SIRT2 activates the p53-p21 pathway through lysine acetylation of p53 in human iPS cells

**Fig. 5:**
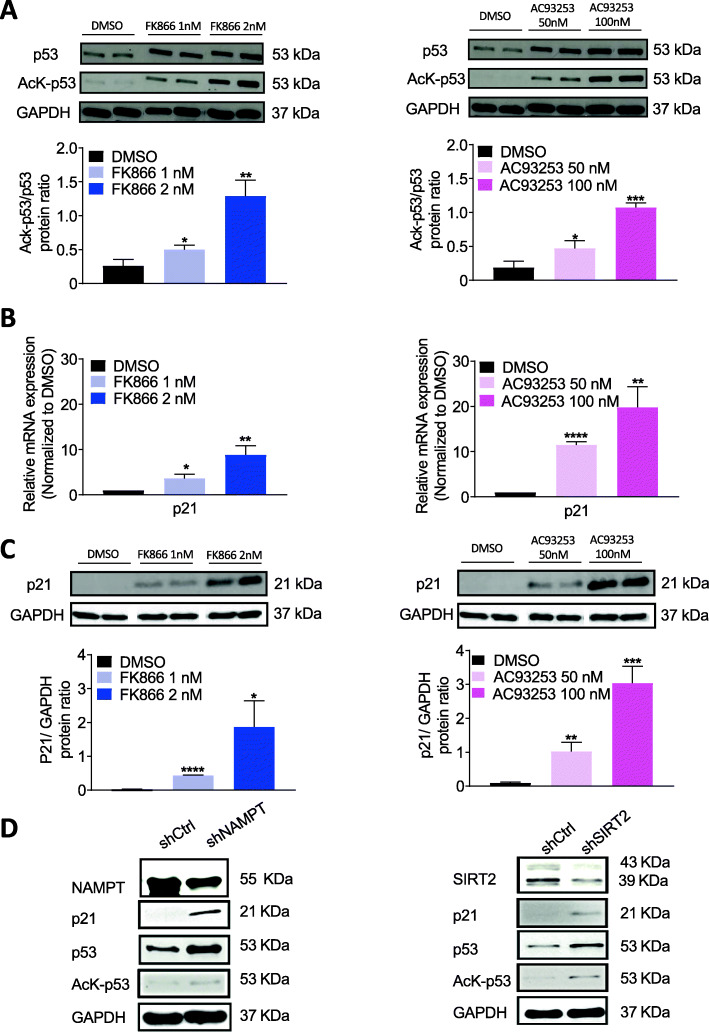
NAMPT or SIRT2 inhibition led to diminished deacetylation of p53 with subsequent upregulation of p21 expression in human iPS cells. **a** Western blot analysis of total p53 and acetyl-K382 p53 protein expression in human iPS cells treated with FK866, AC93253, or DMSO for 48 h. GAPDH was used as loading control. Representative WB images are depicted. Diagrams show acetylated p53 to total p53 protein ratio in arbitrary units (AU). Data represent means ± SD from two independent experiments, each in duplicates (*p < 0.05, **p < 0.01, ***p < 0.001). **b** mRNA expression of p21 in human iPS cells treated with FK866, AC93253, or DMSO for 48 h. Fold changes relative to DMSO-treated cells are shown. Data represent means ± SD from two independent experiments, each in triplicates (*p < 0.05, **p < 0.01, ****p < 0.0001). **c** Western blot analysis of p21 protein expression in human iPS cells treated with FK866, AC93253, or DMSO for 48 h. GAPDH was used as loading control. Representative WB images are depicted. Diagrams show p21 to GAPDH protein ratio in arbitrary units (AU). Data represent means ± SD from two independent experiments, each in duplicates (*p < 0.05, **p < 0.01, ***p < 0.001, ****p < 0.0001). **d** Representative WB images of corresponding protein expression in human iPS cells transduced with the indicated shRNA constructs

We next sought to determine the signaling pathways regulated by NAMPT/SIRT2 in iPS cells. We previously demonstrated the essential role of NAMPT in the regulation of p53 activity in AML cells through lysine-382 deacetylation [11, 21]. p53 plays an important role during the maintenance of iPS cells [22]. In line with these observations, we detected dramatically elevated levels of acetylated and total p53 protein in lysates of FK866- or AC93253-treated iPS cells compared with DMSO-treated samples (Fig. 5a). p53 directly activates p21 [23, 24], which functions as a regulator of cell cycle progression at G1/S phase [25, 26]. Interestingly, p21 mRNA and protein expression were strongly upregulated in response to all tested concentrations of FK866 and AC93253 compared with the DMSO-treated group (Fig. 5b, c).

Inhibition of NAMPT or SIRT2 using shRNA also resulted in the elevated p21 expression due to the activation of p53 by acetylation (Fig. 5d).

**Fig. 6:**
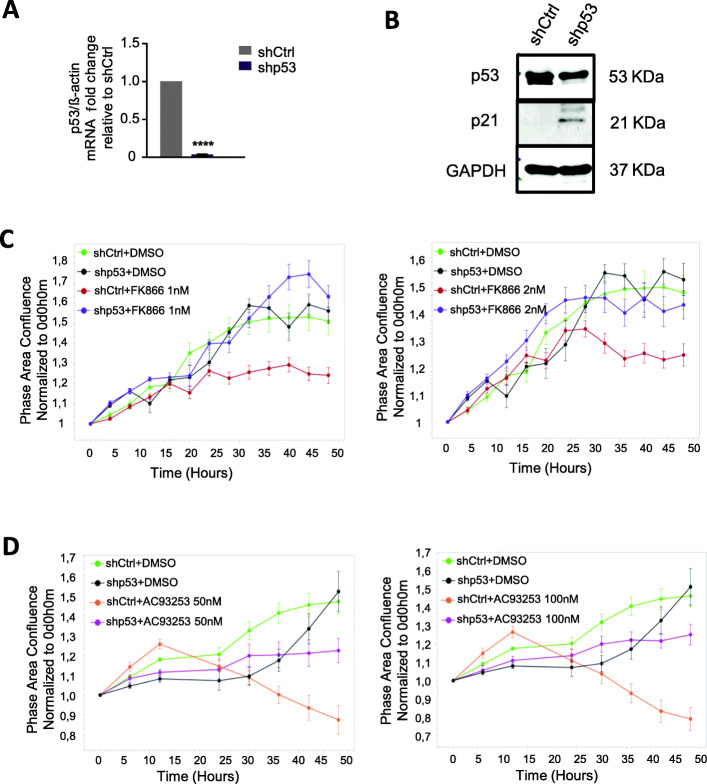
Knockdown of p53 attenuates the growth inhibition in FK866- or AC93253-treated iPSCs. **a** mRNA expression of p53 in iPS cells transduced with shp53 LV was evaluated. iPS cells transduced with shCtrl LV was used as control. Fold change relative to control is shown (****p < 0.0001). **b** Western blot analysis of p53 protein expression in shp53 LV-transduced iPS cells. iPS cells transduced with shCtrl LV was used as control. GAPDH was used as loading control. Representative WB images are depicted. **c**, **d** Growth of iPS cells transduced with control shRNA or p53-specific shRNA and subsequently treated with DMSO, FK866 (1 nM or 2 nM), or AC93253 (50 nM or 100 nM) was evaluated over time with the IncuCyte S3 Live-Cell Analysis System

Moreover, inhibition of p53 using transduction of iPS cells with p53-specific shRNA (Fig. 6a, b) resulted in a markedly diminished reduction of cell proliferation in the presence of NAMPT or SIRT2 inhibitors, as compared to control cells and analyzed using as evaluated using an IncuCyte cell proliferation assay (Fig. 6c, d).

These results suggest that the p53-p21 pathway plays an essential role in NAMPT/SIRT2-mediated proliferation of human iPS cells.

## Discussion

In the present study, we demonstrated for the first time that NAMPT and SIRT2 are indispensable for the maintenance of iPS cells. In doing so, we have opened a new field for further investigation of the role of NAMPT-mediated protein deacetylation in developmental biology. It would be interesting to investigate whether NAMPT-triggered protein deacetylation governs the development of tissues and organs of mesoderm, endoderm or ectoderm origin, and whether SIRT2 or other SIRTs are involved. We recently demonstrated that NAMPT and SIRT2 are essential for early blood cell formation [17]. The results of the present study complemented these findings. Using an EB-based approach, we identified an essential role for NAMPT and SIRT2 in the maintenance of iPS cells and in their hematopoietic differentiation. Until now, nothing was known about the role of NAMPT signaling in endodermal or ectodermal cell specification. Here, we found deregulated expression of genes specific for the three germ layers. We also observed no effects of NAMPT or SIRT2 inhibition on the ectodermal differentiation, but strong effect on the endodermal and mesodermal differentiation. Our observations may help to identify improved culture conditions through pharmacological modulation of NAMPT/SIRT signaling, extending existing novel strategies for in vitro growth of different tissues as organoid cultures for translational and even therapeutic use. Our observations suggest that addition of NAMPT, NA, or NAD^+^ may be useful for the maintenance or generation of iPS cells. Moreover, knowledge about the essential role of NAMPT/SIRT2 signaling for the maintenance of pluripotency in iPS cells may suggest better screening strategies for distinguishing pluripotent, high-quality iPS cells from differentiated, low-quality iPS cells based on an analysis of NAMPT/SIRT2 pathway activity. In addition, by modulating NAMPT/SIRT2 signaling in iPS cell culture, we may improve the quality of generated iPS cells. Our findings also suggest that NAMPT/SIRT2 signaling regulates iPS cell proliferation, an observation that may help in generating iPS cells more efficiently for large-scale culture.

The effects of NAMPT or SIRTs on the specification and differentiation of tissues and organs may be cell type-, concentration-, and differentiation stage-dependent. Dose- and cell type-dependent functions of NAMPT are known: although NAMPT is required for proper myeloid cell formation, hyper-activated NAMPT induces proliferation of hematopoietic stem cells and causes acute myeloid leukemia [9]. In the present study, we demonstrated that inhibition of NAMPT in iPS cells abrogated early-stage hematopoietic differentiation, arguing for a pro-differentiation role of NAMPT in this cell type. It is known that a SIRT1 deficiency causes increased apoptosis and diminished hematopoietic differentiation of mouse ES cells [14, 27]. We found here that SIRT2 also plays a role in apoptosis induction and/or hematopoietic differentiation of iPS cells. We previously reported that SIRT2 inhibition resulted in apoptosis and diminished proliferation of acute myeloid leukemia cells through deacetylation/activation of Akt and subsequent activation of ß-catenin [9]. Both Akt and ß-catenin are important regulators of the proliferation of iPS cells and hematopoietic stem cells. But whether SIRT2 is connected to ß-catenin and Akt activation in the maintenance of the pluripotent state of iPS cells or induction of the mesodermal stages of hematopoiesis remains to be investigated.

A search for downstream targets of NAMPT/SIRT2 revealed that p53 is deacetylated by NAMPT/SIRT2 in iPS cells, leading to rapid and robust activation of p21 with subsequent cell cycle arrest and apoptosis of iPS cells. We recently reported that NAMPT deacetylates p53 in myeloid leukemia cells [11]. Our findings in iPS cells further confirm the important role of NAMPT/SIRT2 in p53 deactivation through lysine deacetylation. It has been reported that the p53-p21 pathway functions as a barrier that inhibits iPS cell generation and that p53 deletion improves the efficiency of iPS cell generation [28]. In this context, suppression of p53 activity through pharmacological modulation of the NAMPT/SIRT2 pathway may improve the efficiency of iPS cell generation. It would be interesting to investigate whether the p53-p21 pathway is connected to the ß-catenin-Akt pathway in iPS cells. The ultimate mechanism by which NAMPT and SIRT2 contribute to the regulation of hematopoietic differentiation also needs to be studied.

NAD^+^ may not only regulate NAMPT/SIRT-triggered protein deacetylation, it may also affect intracellular metabolic processes. Metabolic regulation of iPS cells during reprogramming and self-renewal has recently been described [29, 30]. In line with these observations, it would be interesting to investigate the effect of NAMPT inhibition on the metabolic processes operating during iPS cell maintenance or hematopoietic differentiation. This may lead to the identification of novel small molecules connected to NAD^+^ that are capable of inducing pluripotency or hematopoietic differentiation of iPS cells.

Taken together, our findings describe a novel mechanism of iPS cell maintenance by NAMPT/SIRT2 through post-translational regulation of p53. This mechanism is of central importance for developmental biologists, cell biologists, and clinical scientists.

## Conclusions

Identification of the novel mechanisms essential for the proper proliferation and hematopoietic differentiation of iPS cells might lead to an establishment of novel improved protocols for the iPS cell maintenance and high-scale production for clinical use. We described here the novel role of the NAMPT/SIRT2 pathway in the regulation of p53 activation by deacetylation. We demonstrated that NAMPT or SIRT2 inhibition resulted in the strong induction of p21 expression that ultimately led to the cell cycle arrest, apoptosis, and disturbed differentiation of iPS cells.

### Supplementary Information

**Additional file 1.**

**Additional file 2.**

**Additional file 3.**

## Data Availability

Supporting data are available online.

## Abbreviations

NAMPT: Nicotinamide phosphoribosyltransferase
iPS: Induced pluripotent stem
EB: Embryoid body
ES: Embryonic stem
NAD: Nicotinamide adenine dinucleotide
DMSO: Dimethylsulfoxide
DAPI: 4′,6-Diamidino-2-phenylindole
FACS: Fluorescence-activated cell sorting
qPCR: Quantitative polymerase chain reaction
PBS: Phosphate-buffered saline
TBST: Tris buffered saline with Tween-20
NBT/BCIP: 5-Bromo-4-chloro-3-indolyl phosphate/nitro blue tetrazolium
PFA: Paraformaldehyde
HRP: Horseradish peroxidase
ECL: Enhanced chemiluminescence
BrdU: Bromodeoxyuridine / 5-bromo-2'-deoxyuridine
EDTA: Ethylenediaminetetraacetic acid
G-CSF: Granulocyte-colony stimulating factor
CRISPR: Clustered Regularly Interspaced Short Palindromic Repeats
PAX6: Paired Box 6
TUB3: Alpha-tubulin
MYH6: Myosin Heavy Chain 6
FOXA2: Forkhead Box A2
AFP: Alpha-fetoprotein
