# Correction: NAMPT/SIRT2-mediated inhibition of the p53-p21 signaling pathway is indispensable for maintenance and hematopoietic differentiation of human iPS cells

**DOI:** 10.1186/s13287-023-03511-4

**Published:** 2023-10-20

**Authors:** Yun Xu, Masoud Nasri, Benjamin Dannenmann, Perihan Mir, Azadeh Zahabi, Karl Welte, Tatsuya Morishima, Julia Skokowa

**Affiliations:** 1 Department of Hematology, Oncology, Clinical Immunology and Rheumatology, University Hospital Tübingen, Tübingen, Germany; 2 University Children’s Hospital Tübingen, Tübingen, Germany; 3 Present Address: International Research Center for Medical Sciences, Kumamoto University, Kumamoto, Japan

**Correction: Stem Cell Research & Therapy (2021) 12:112** 10.1186/s13287-021-02144-9

1. In the original Fig. 5A, C the order of the samples was reorganized by cutting the bands from the original blot and putting them in the sequence DMSO, FK866 1nM, and FK866 2nM from left to right, but the vertical lines indicating the cutting positions of the blot were missing.
  We have now replaced Fig. 5A, C with the original uncut Western Blot images. Note that the bands marked with the asterisk were excluded from the densitometry analysis of presented Western Blot images due to the poor protein quality of the sample, as indicated by the weak GAPDH signal. For the statistical analysis of the FK866 2nM datasets, we accidentally calculated the *p*-value of two datasets only instead of the required three, as was calculated for the DMSO and FK866 1nM datasets, respectively. Therefore, we removed the stars indicating the significance of the FK866 2nM datasets from the corresponding densitometry graphs, which doesn’t change the conclusion, because the difference between DMSO and also FK866 2nM treated samples is obvious. We also corrected legends for Fig. 5A, C regarding the analyzed datasets of the presented WB images, for which statistics were performed.
**Corrected figure legend**
**Figure 5**
**a** Western blot analysis of total p53 and acetyl-K382 p53 protein expression in human iPS cells treated with FK866, AC93253, or DMSO for 48 h. GAPDH was used as a loading control. Representative WB images are depicted. The bands marked with the asterisk were excluded from the densitometry analysis due to the poor protein quality of the sample. Diagrams show the acetylated p53 to total p53 protein ratio in arbitrary units (AU). Data represent means ± SD (n = 2–3) (*p < 0.05, ***p < 0.001).
**c** Western blot analysis of p21 protein expression in human iPS cells treated with FK866, AC93253, or DMSO for 48 h. GAPDH was used as a loading control. Representative WB images are depicted. The bands marked with the asterisk were excluded from the analysis due to the bad protein quality of the sample. Diagrams show p21 to GAPDH protein ratio in arbitrary units (AU). Data represent means ± SD (n = 2–3) (**p < 0.01, ***p < 0.001, ****p < 0.0001)
In the original Fig. 5D, the GAPDH bands as loading controls for immune detection of p53, AcK-p53, and p21 were missing. We have now included the missing GAPDH bands.
In the original Fig. 6B, the line indicating the cutting position of the p53 blot was missing. We have now included this line.

None of the aforementioned corrections affect the conclusions of the respective experiments.

