# Supplementary material for: NAMPT/SIRT2-mediated inhibition of the p53-p21 signaling pathway is indispensable for maintenance and hematopoietic differentiation of human iPS cells
Source: Stem Cell Res Ther. 2021 Feb 5;12:112. doi: 10.1186/s13287-021-02144-9 (PMC7863436; doi:10.1186/s13287-021-02144-9)
Supplement: Supplementary file 1 — Additional file 1. [file 13287_2021_2144_MOESM1_ESM.docx]

**Table S1. qRT-PCR primer sequences**

|  | **Forward primer (5'-3')** | **Reverse primer (5'-3')** |
| --- | --- | --- |
| **GAPDH** | CTGGGCTACACTGAGCACC | AAGTGGTCGTTGAGGGCAATG |
| **OCT4** | CCTCACTTCACTGCACTGTA | CAGGTTTTCTTTCCCTAGCT |
| **SOX2** | TTCACATGTCCCAGCACTACCAGA | TCACATGTGTGAGAGGGGCAGTGTGC |
| **NANOG** | RVGAACACAGTTCTGGTCTTCTG | WTCACACGGAGACTGTCTCTC |
| **DNMT** | ATAAGTCGAAGGTGCGTCGT | GGCAACATCTGAAGCCATTT |
| **PAX6** | ACCCATTATCCAGATGTGTTTGCCCGAG | ATGGTGAAGCTGGGCATAGGCGGCAG |
| **TUB3** | TAGACCCCAGCGGCAACTAT | GTTCCAGGTTCCAAGTCCACC |
| **MYH6** | GCCCTTTGACATTCGCACTG | GGTTTCAGCAATGACCTTGCC |
| **BRACH** | CTGGGTACTCCCAATGGGG | GGTTGGAGAATTGTTCCGATGA |
| **FOXA2** | TGGGAGCGGTGAAGATGGAAGGGCAC | TCATGCCAGCGCCCACGTACGACGAC |
| **AFP** | GAATGCTGCAAACTGACCACGCTGGAAC | TGGCATTCAAGAGGGTTTTCAGTCTGGA |
| **TP53** | GCCCAACAACACCAGCTCCT | CCTGGGCATCCTTGAGTTCC |
| **P21** | TGTCCGTCAGAACCCATGC | AAAGTCGAAGTTCCATCGCTC |
| **NAMPT** | GCAGAAGCCGAGTTCAACATC | TGCTTGTGTTGGGTGGATATTG |
| **ß-actin** | AGCGGGAAATCGTGCGTG | GGGTACATGGTGGTGCCG |
