# Supplementary material for: NAMPT/SIRT2-mediated inhibition of the p53-p21 signaling pathway is indispensable for maintenance and hematopoietic differentiation of human iPS cells
Source: Stem Cell Res Ther. 2021 Feb 5;12:112. doi: 10.1186/s13287-021-02144-9 (PMC7863436; doi:10.1186/s13287-021-02144-9)
Supplement: Supplementary file 2 — Additional file 2. [file 13287_2021_2144_MOESM2_ESM.pdf]

A

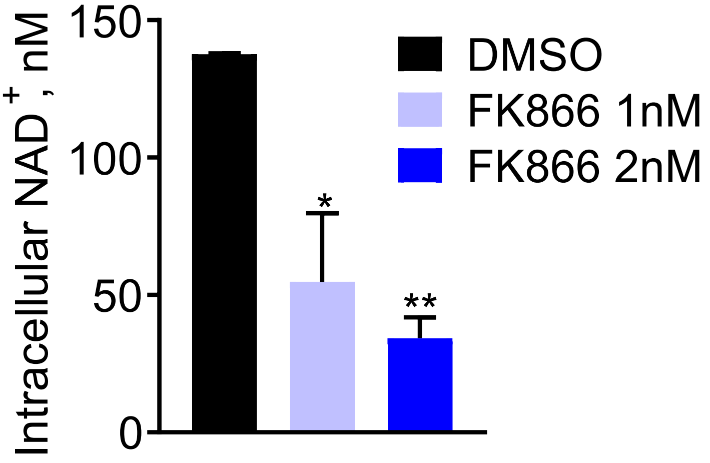

Figure S1

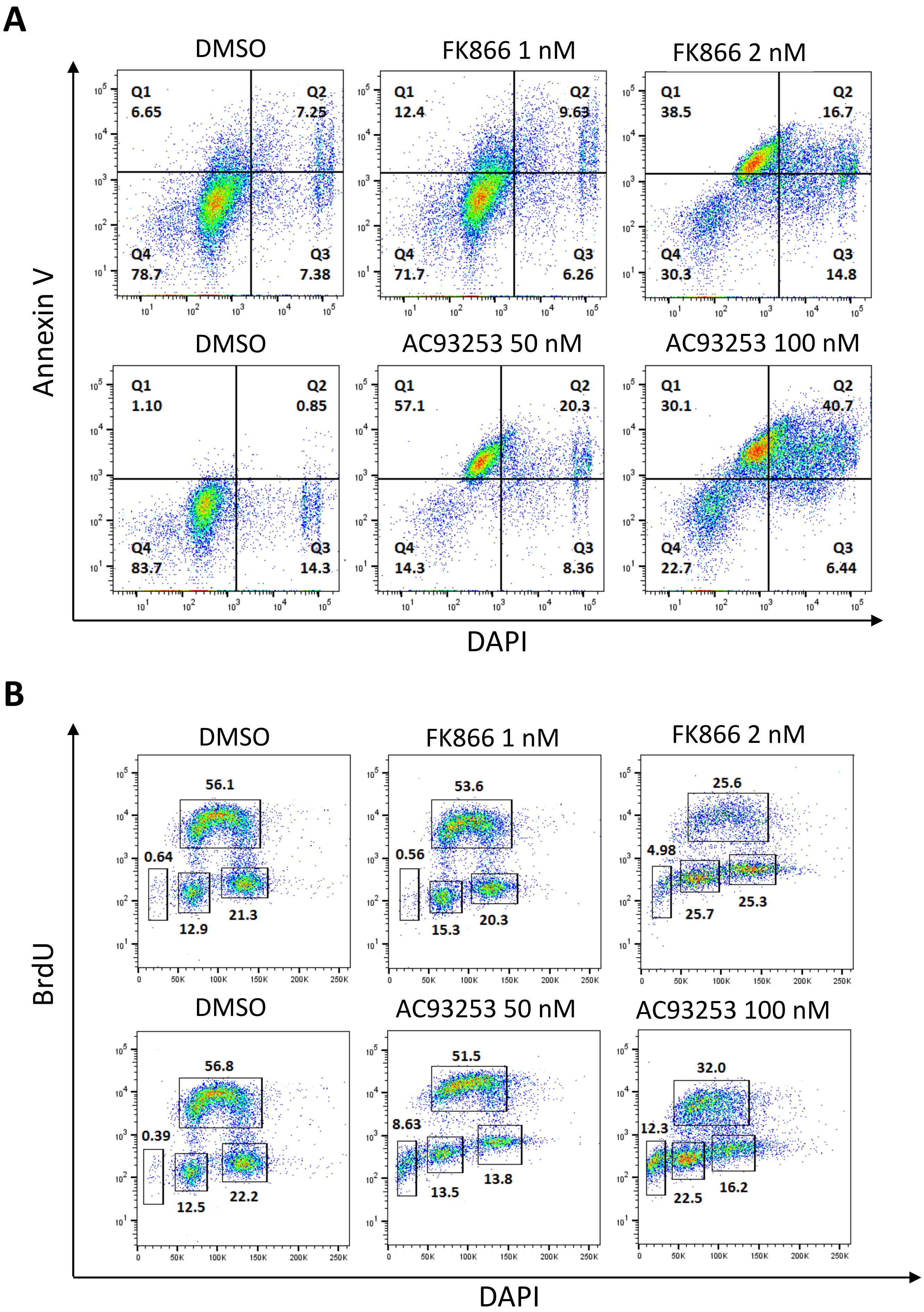
A
DMSO
FK866 1 nM
FK866 2 nM
Q1 6.65
Q2 7.25
Q4 78.7
Q3 7.38
Q1 12.4
Q2 9.63
Q4 71.7
Q3 6.26
Q1 38.5
Q2 16.7
Q4 30.3
Q3 14.8
DMSO
AC93253 50 nM
AC93253 100 nM
Q1 1.10
Q2 0.85
Q4 83.7
Q3 14.3
Q1 57.1
Q2 20.3
Q4 14.3
Q3 8.36
Q1 30.1
Q2 40.7
Q4 22.7
Q3 6.44
Annexin V
DAPI
B
DMSO
FK866 1 nM
FK866 2 nM
56.1
0.64
12.9
21.3
53.6
0.56
15.3
20.3
25.6
4.98
25.7
25.3
DMSO
AC93253 50 nM
AC93253 100 nM
56.8
0.39
12.5
22.2
51.5
8.63
13.5
13.8
32.0
12.3
22.5
16.2
BrdU
DAPI

Figure S2

A

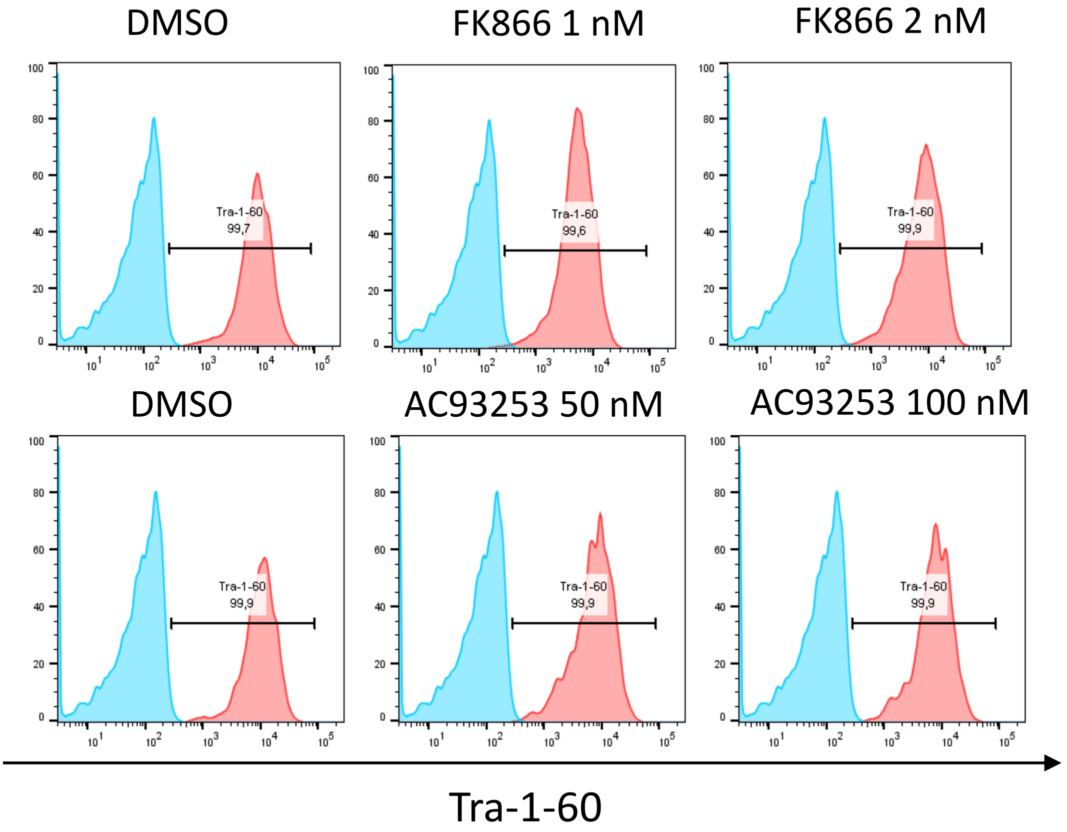

DMSO
FK866 1 nM
FK866 2 nM
Tra-1-60
99,7
99,6
99,9
DMSO
AC93253 50 nM
AC93253 100 nM
Tra-1-60
99,9
99,9
99,9
Tra-1-60

B

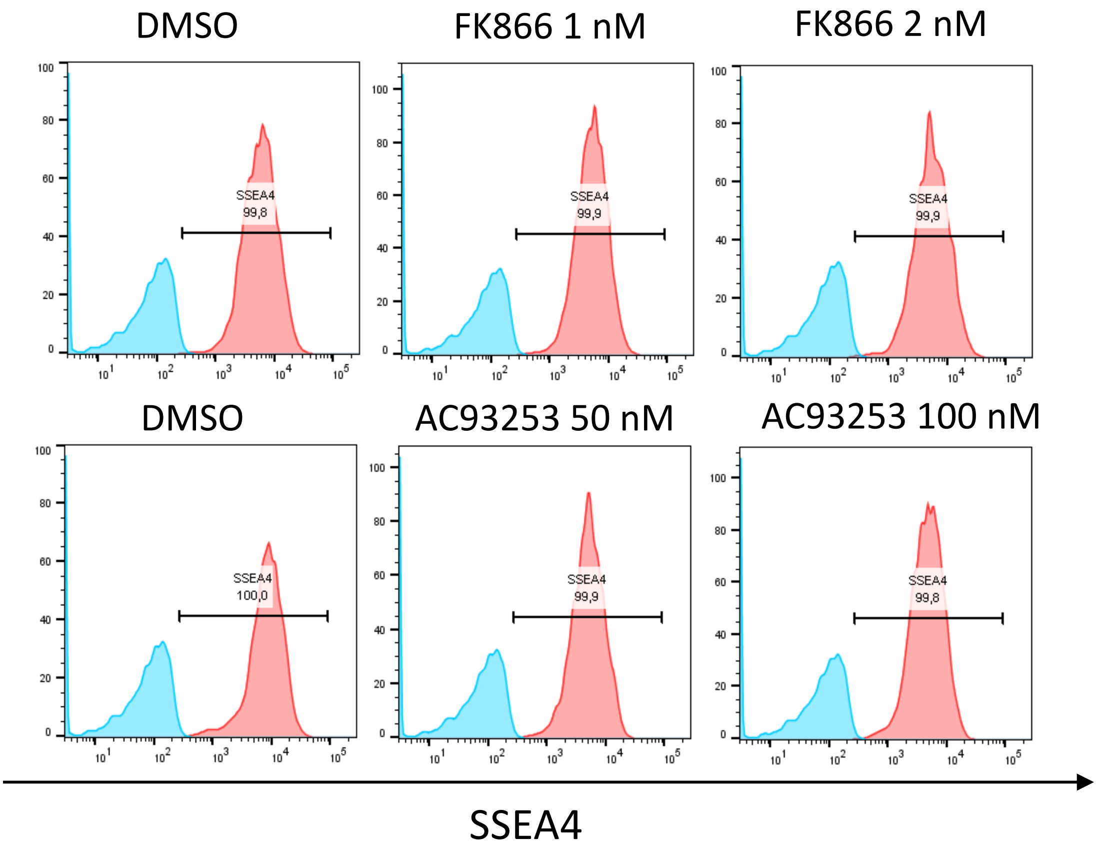

DMSO
FK866 1 nM
FK866 2 nM
SSEA4
99,8
99,9
99,9
DMSO
AC93253 50 nM
AC93253 100 nM
SSEA4
100,0
99,9
99,8
SSEA4

Figure S3

A

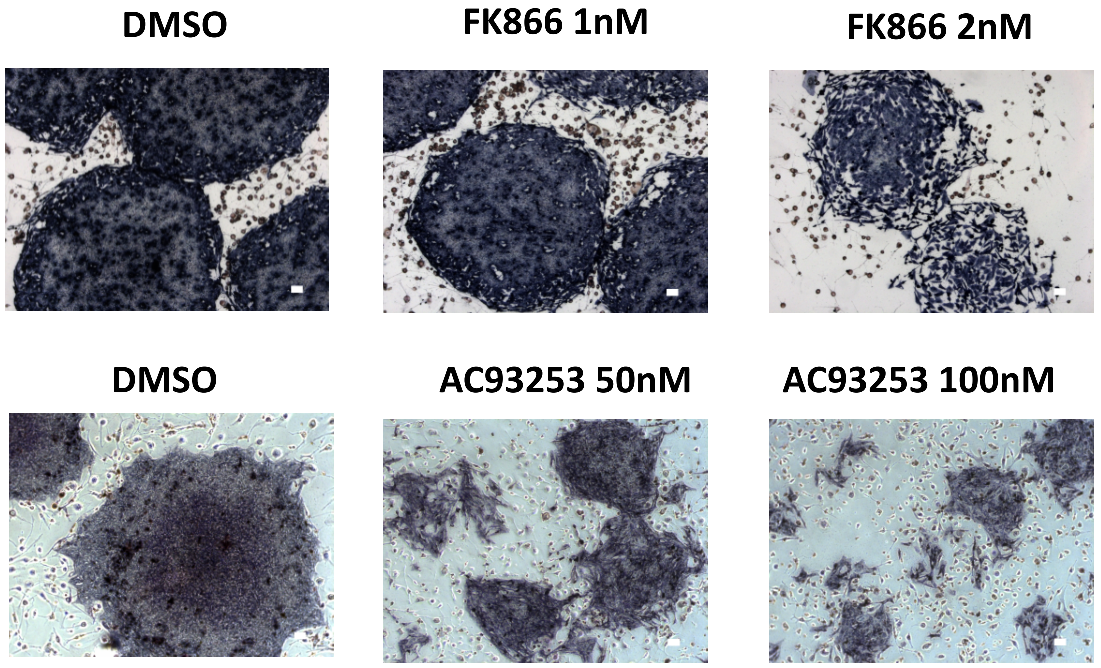

Figure S4

A

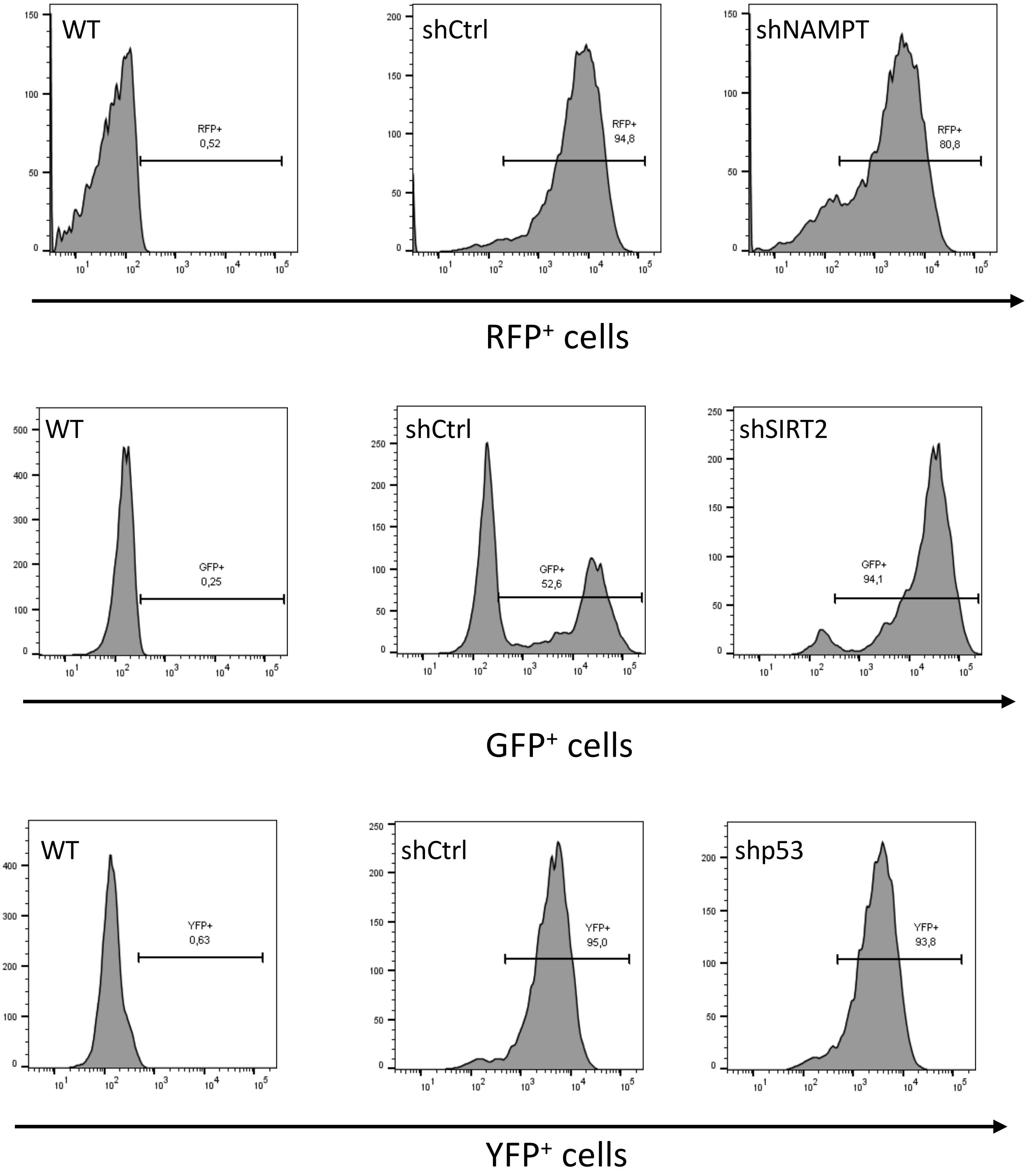
WT
RFP+
0,52
shCtrl
RFP+
94,8
shNAMPT
RFP+
80,8
RFP+ cells
WT
GFP+
0,25
shCtrl
GFP+
52,6
shSIRT2
GFP+
94,1
GFP+ cells
WT
YFP+
0,63
shCtrl
YFP+
95,0
shp53
YFP+
93,8
YFP+ cells

Figure S5

A

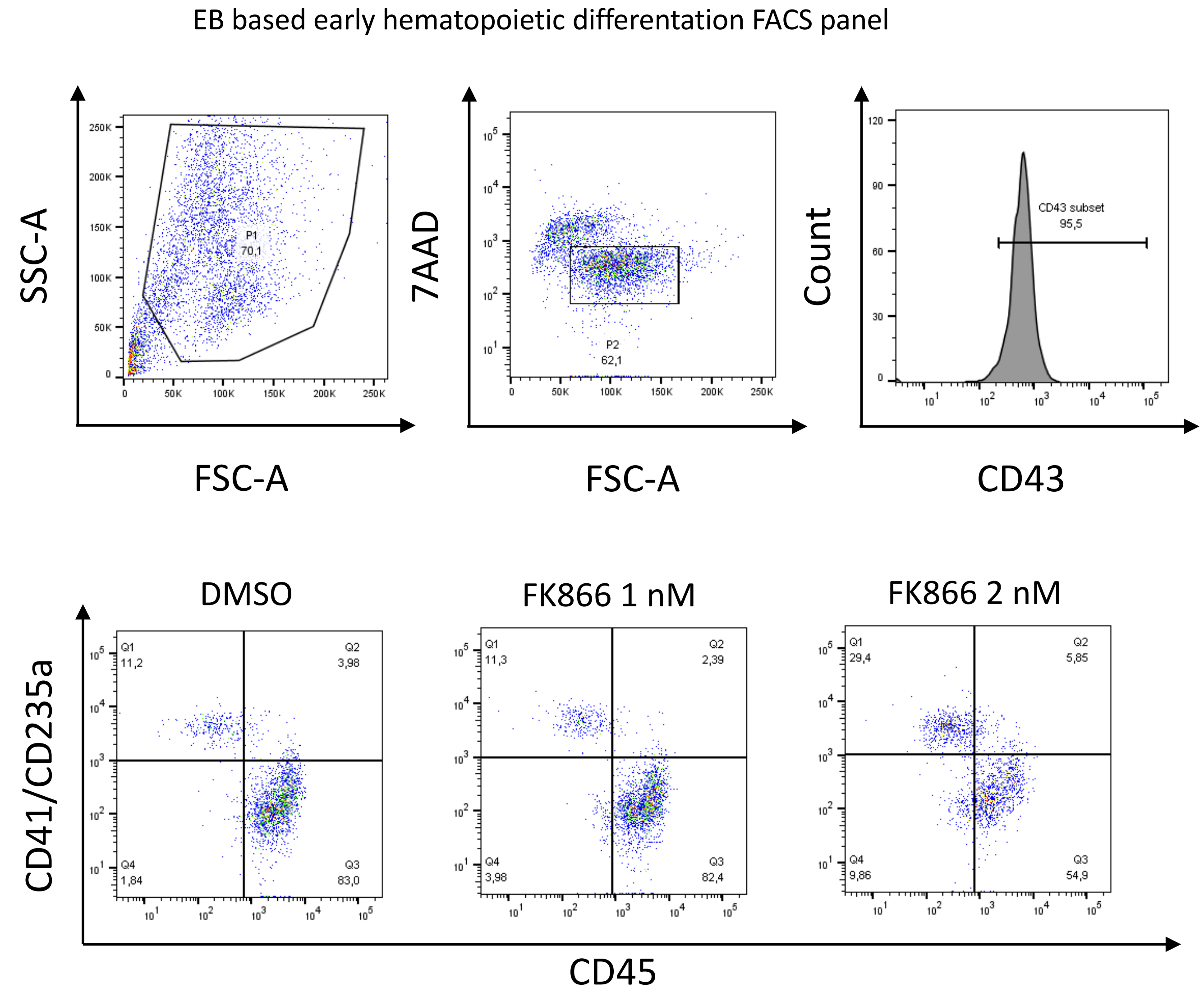

EB based early hematopoietic differentation FACS panel
SSC-A
FSC-A
P1
70,1
7AAD
P2
62,1
Count
CD43
CD43 subset
95,5
DMSO
FK866 1 nM
FK866 2 nM
CD41/CD235a
CD45
Q1 11,2
Q2 3,98
Q4 1,84
Q3 83,0
Q1 11,3
Q2 2,39
Q4 3,98
Q3 82,4
Q1 29,4
Q2 5,85
Q4 9,86
Q3 54,9

Figure S6

A

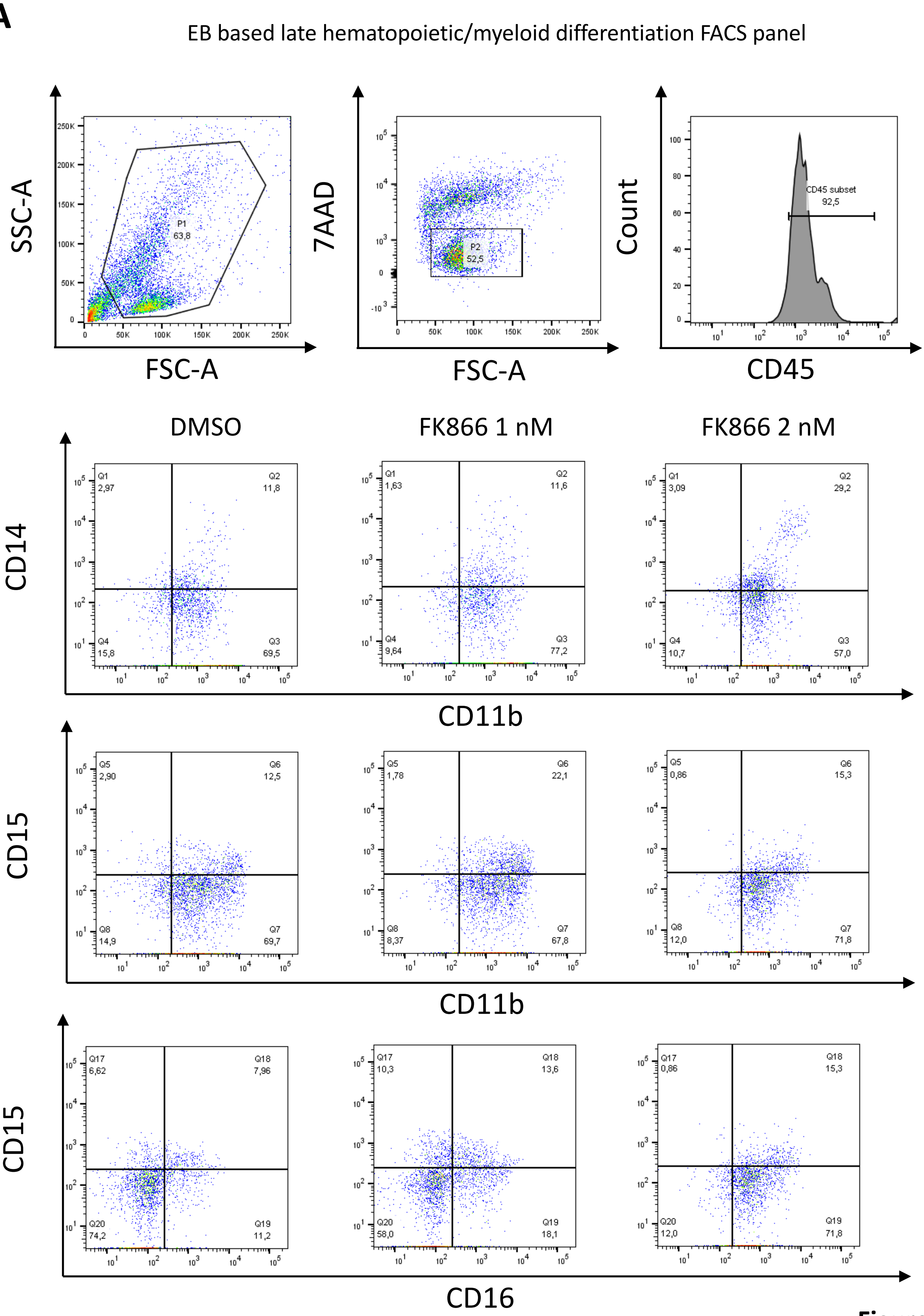

EB based late hematopoietic/myeloid differentiation FACS panel
SSC-A
FSC-A
P1
63,8
7AAD
P2
52,5
Count
CD45 subset
92,5
CD45
DMSO
FK866 1 nM
FK866 2 nM
CD14
CD11b
Q1 2,97
Q2 11,8
Q4 15,8
Q3 69,5
Q1 1,63
Q2 11,6
Q4 9,64
Q3 77,2
Q1 3,09
Q2 29,2
Q4 10,7
Q3 57,0
CD15
CD11b
Q5 2,90
Q6 12,5
Q8 14,9
Q7 69,7
Q5 1,78
Q6 22,1
Q8 8,37
Q7 67,8
Q5 0,86
Q6 15,3
Q8 12,0
Q7 71,8
CD15
CD16
Q17 6,62
Q18 7,96
Q20 74,2
Q19 11,2
Q17 10,3
Q18 13,6
Q20 58,0
Q19 18,1
Q17 0,86
Q18 15,3
Q20 12,0
Q19 71,8

Figure S7

A

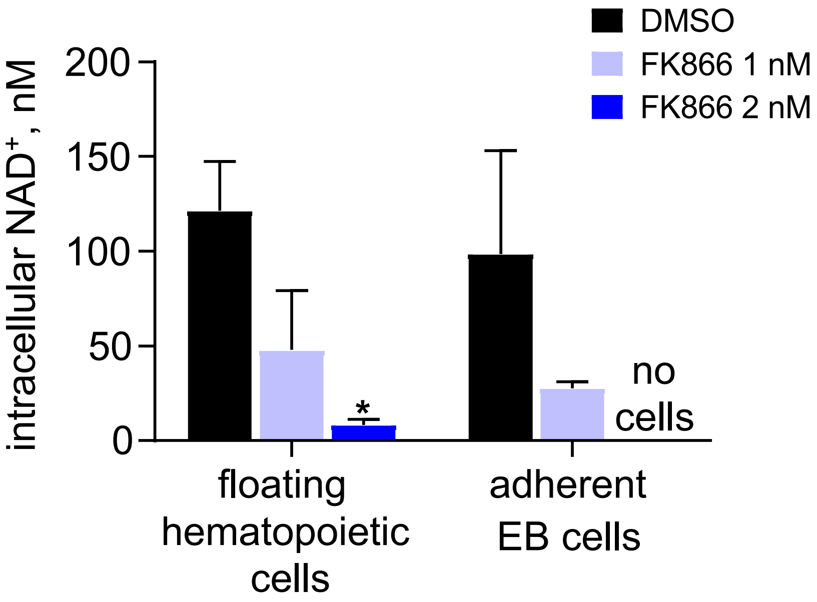

DMSO
FK866 1 nM
FK866 2 nM
intracellular NAD+, nM
200
150
100
50
0
*
no cells
floating hematopoietic cells
adherent EB cells

Figure S8
