# Supplementary material for: NAMPT/SIRT2-mediated inhibition of the p53-p21 signaling pathway is indispensable for maintenance and hematopoietic differentiation of human iPS cells
Source: Stem Cell Res Ther. 2021 Feb 5;12:112. doi: 10.1186/s13287-021-02144-9 (PMC7863436; doi:10.1186/s13287-021-02144-9)
Supplement: Supplementary file 3 — Additional file 3. [file 13287_2021_2144_MOESM3_ESM.docx]

**NAMPT/SIRT2–mediated inhibition of the p53-p21 signaling pathway is indispensable for maintenance and hematopoietic differentiation of human iPS cells**

Yun Xu^1^, Masoud Nasri^1^, Benjamin Dannenmann^1^, Perihan Mir^1,2^, Azadeh Zahabi^1^, Karl Welte^4^, Tatsuya Morishima^1,*, #,§^, Julia Skokowa^1,*, #^

**Supplemental information**

**Supplemental methods**

**Intracellular NAD^+^ measurement**

Intracellular NAD^+^ was measured in cell lysates of 1 × 10^4^ cells using the NAD/NADH-Glo™ Assay Kit (Promega). Luminescence was detected with a GloMax®-Multi+ Detection System (Promega).

**Alkaline phosphatase staining**

iPS cells in cell culture dishes were washed once with PBS, fixed with 4% PFA (Cat Nr. P6148-500G, Sigma-Aldrich) for 2 minutes at room temperature and washed twice with PBS. After that, cells were incubated with NBT/BCIP dye (Cat Nr. 72091-10ML, Sigma-Aldrich) for 20 minutes at room temperature in a dark and washed once with PBS. Images were taken on the Nikon Eclipse TS 100 microscope.

**Supplemental figures**

**Figure S1. Inhibition of NAMPT suppresses intracellular NAD^+^ levels in human iPS cells**

(A) Intracellular NAD^+^ levels in human iPS cells treated with different concentration of FK866 for 48 hours. The same concentration of DMSO was added as a vehicle control. Data represent means ± SD from three independent experiments, each in triplicate (**p* < 0.05, ***p* < 0.01 compared to DMSO-treated cells).

**Figure S2. Inhibition of NAMPT/SIRT2 pathway induces apoptosis and cell cycle arrest in human iPS cells**

(A, B) Annexin V staining (A) and cell cycle analysis using BrdU assay (B) in human iPS cells treated with indicated drugs for 48 hours. The same concentration of DMSO was added as a vehicle control. Representative flow cytometry dot plot images are shown.

**Figure S3. Tra-1-60 and SSEA4 surface expression on human iPS cells treated with NAMPT- or SIRT2 inhibitors**

(A, B) Flow cytometry analysis of TRA-1-60 (A) and SSEA4 (B) surface expression on human iPS cells treated with indicated drugs for 48 hours. The same concentration of DMSO was added as a vehicle control. Representative flow cytometry histogram images are shown.

**Figure S4. Inhibition of NAMPT or SIRT2 does not affect AP activity in iPS cells**

(A) Alkaline phosphatase activity in human iPS cells treated with indicated drugs for 48 hours. Representative images are shown. Images were taken using Nikon Eclipse TS 100 microscope. Scale bars: 50 µm.

**Figure S5. Transduction efficiency of iPS cells with different shRNA constructs**

(A) Flow cytometry analysis of RFP^+^, GFP^+^ and YFP^+^ iPS cells transduced with corresponding shRNA constructs. FACS analysis was performed XXX hours after transduction. Representative FACS histogram images are shown.

**Figure S6. The effects of NAMPT- and SIRT2 inhibition on the early stages of EB-based hematopoietic differentiation of iPS cells**

(A) Representative FACS dot plot and histogram images showing the gating strategy of the analysis of EB based hematopoietic/myeloid differentiation at the early stage (day18 of culture).

**Figure S7. The effects of NAMPT- and SIRT2 inhibition on the late stages of EB-based hematopoietic/myeloid differentiation of iPS cells**

(A) Representative FACS dot plot and histogram images showing the gating strategy of the analysis of EB based hematopoietic/myeloid differentiation at the late stage (day 25 of culture).

**Figure S8. Intracellular NAD^+^ levels in EBs and in floating hematopoietic cells derived from human iPS cells in EB-based hematopoietic differentiation culture**

(A) Intracellular NAD^+^ levels in corresponding cell populations were assessed on day 29 of EB-based hematopoietic differentiation culture in the presence of different concentration of FK866. Floating hematopoietic cells and adherent cells of embryoid bodies were collected and analyzed separately. The same concentration of DMSO was added as a vehicle control. Data represent means ± SD from three independent experiments, each in triplicate (**p* < 0.05 compared to DMSO-treated cells).
